# Taxifolin protects against doxorubicin-induced cardiotoxicity and ferroptosis by adjusting microRNA-200a-mediated Nrf2 signaling pathway

**DOI:** 10.1016/j.heliyon.2023.e22011

**Published:** 2023-11-08

**Authors:** Zhihui Lin, Jie Wang

**Affiliations:** a Department of Endocrinology, The Second Affiliated Hospital and Yuying Children's Hospital, Wenzhou Medical University, Wenzhou, 325000, Zhejiang, China; b Department of Cardiology, The Second Affiliated Hospital and Yuying Children's Hospital, Wenzhou Medical University, Wenzhou, 325000, Zhejiang, China

**Keywords:** Taxifolin, Doxorubicin, miR-200a, Nrf2, Ferroptosis, Cardiotoxicity

## Abstract

The chemotherapeutic agent doxorubicin (Dox) is commonly used to treat various types of cancer, even though it can cause life-threatening cardiotoxicity. Clinically, there is no particularly effective way to treat Dox-induced cardiotoxicity. Therefore, it is imperative to identify compounds that can effectively alleviate Dox-induced cardiotoxicity. Ferroptosis and oxidative stress play a key role in Dox-induced cardiotoxicity, and the inhibition of ferroptosis and oxidative stress could effectively protect against doxorubicin-induced cardiotoxicity. Taxifolin (TAX) is a flavonoid commonly found in onions and citrus fruits. In the present study, we evaluated the effects of TAX on Dox-induced cardiac injury and dysfunction and aimed to explore the mechanisms underlying these effects. Using a mouse model of Dox-induced cardiotoxicity, we administered 20 mg/kg/day of TAX by gavage for 2 weeks. A week after the first use of TAX, each mouse was administered a 10 mg/kg dose of Dox. TAX was first evaluated for its cardioprotective properties, and the outcomes showed that TAX significantly reduced the damage caused by Dox to the myocardium in terms of structural and functional damage by effectively inhibiting ferroptosis and oxidative stress. In vivo, echocardiography, histopathologic assay, serum biochemical analysis and western blotting was used to find the results that Dox promoted ferroptosis-induced cardiomyocyte death, while TAX reversed these effects. In vitro, we also found that TAX alleviated Dox-induced cardiotoxicity by using ROS/DHE staining assay, Cellular immunofluorescence and western blotting. TAX increasing expression of microRNA-200a (miR-200a) which affects ferroptosis by activating Nrf2 signaling pathway. We believe that TAX inhibits ferroptosis and is a potential phytochemical that prevents Dox-induced cardiotoxicity.

## 1 Introduction

Doxorubicin (Dox) is a chemotherapy medication commonly used to treat various types of cancer, including breast cancer, ovarian cancer, and leukemia [1,2]. It works by inhibiting the growth of cancer cells by interfering with the DNA synthesis process [3]. However, despite its effectiveness in treating cancer, Dox has significant side effects, such as cardiotoxicity, which can lead to irreversible damage to the heart muscle [[4], [5], [6]]. Therefore, careful monitoring and management of patients undergoing Dox treatment is necessary to minimize the risk of adverse effects.

One of the mechanisms of Dox-induced cardiotoxicity is believed to be related to the overproduction of reactive oxygen species (ROS) and oxidative stress-induced mitochondrial damage [[7], [8], [9], [10]]. ROS can cause oxidative damage to biological macromolecules, including lipids, proteins, and DNA, which can disrupt cellular membrane integrity and function [11,12]. Therefore, strategies to reduce oxidative stress and protect against mitochondrial damage are being explored to minimize the risk of cardiotoxicity in patients undergoing Dox treatment.

Ferroptosis is a specific form of cell death that is triggered by iron-dependent lipid peroxidation [13,14]. Recent studies have suggested that ferroptosis may also play a role in Dox-induced cardiotoxicity [15]. Dox can induce the accumulation of iron in cardiac cells, which can trigger ferroptosis [16]. In this process, iron ions accumulate within the cell and react with free radicals such as hydrogen peroxide to form highly reactive hydroxyl radicals, leading to increased levels of oxidative stress within the cell [17]. As oxidative stress intensifies, the cell eventually enters a state of ferroptosis. Therefore, in addition to ROS and mitochondrial damage, ferroptosis may also be an important mechanism underlying Dox-induced cardiotoxicity. Researchers are exploring ways to inhibit the ferroptosis pathway to mitigate the cardiotoxic effects of Dox. This may involve the use of antioxidants or iron chelators, which can reduce the accumulation of iron and prevent lipid peroxidation. Nrf2 (Nuclear factor erythroid 2-related factor 2) is a transcription factor that plays a key role in regulating the antioxidant response in cells [18,19]. When cells are exposed to oxidative stress or other environmental stressors, Nrf2 is activated and moves into the nucleus of the cell, where it binds to DNA and induces the expression of genes that encode antioxidant and detoxification enzymes [20]. The activation of Nrf2 is essential for protecting cells from oxidative damage and promoting cell survival. However, the dysregulation of Nrf2 activity has also been implicated in the development of various diseases, including cancer, neurodegenerative disorders, and metabolic diseases [21]. The Keap1-Nrf2 pathway is a key mechanism for regulating the cellular response to oxidative stress and other environmental stressors [22]. Keap1 acts as a cytoplasmic sensor that binds to Nrf2 and targets it for degradation via the ubiquitin-proteasome system [23]. However, under conditions of oxidative stress, Nrf2 is stabilized and accumulates in the cytoplasm due to modifications of Keap1 that disrupt its ability to target Nrf2 for degradation [24]. This allows Nrf2 to translocate into the nucleus and activate the transcription of genes that encode antioxidant and detoxification enzymes, as well as other cytoprotective proteins [25].

MiR-200a is a small non-coding RNA molecule that is involved in post-transcriptional regulation of gene expression and be used as possible therapeutic targets to combat disease [26]. It belongs to the miRNA-200 family, which is involved in various biological processes, such as cell differentiation, proliferation, and apoptosis [27]. MiR-200a has been found to play important roles in cancer progression, including tumor growth, invasion, and metastasis. It targets several genes involved in these processes, including E-cadherin, ZEB1, and ZEB2 [28]. Dysregulation of miR-200a has been associated with various types of cancer, such as breast cancer, ovarian cancer, and lung cancer [29]. Additionally, miR-200a led to Nrf2 activation as it targeted Keap1 mRNA and promoted its degradation in heart failure with preserved ejection fraction [30].

Taxifolin, also known as dihydroquercetin, is a flavonoid compound found in various plants, including conifers, ginkgo biloba, and milk thistle [31]. It is a type of flavonoid called a flavanone and is closely related to the more well-known flavonoid quercetin. Taxifolin has been shown to have a range of potential health benefits, including antioxidant, anti-inflammatory, and anti-cancer properties [32,33]. It has also been investigated for its potential to improve cardiovascular health, reduce the risk of diabetes and metabolic disorders, and enhance cognitive function [34]. Studies have suggested that taxifolin may help to protect against oxidative stress by scavenging free radicals and reducing lipid peroxidation. It may also have anti-inflammatory effects by inhibiting the production of pro-inflammatory cytokines. In addition, taxifolin has been shown to have potential anti-cancer properties, with some studies suggesting that it may inhibit the growth and proliferation of cancer cells. It may also enhance the efficacy of chemotherapy drugs and reduce their toxic side effects [35,36]. The current study examined the significant mitigating effect of Taxifolin on Dox-induced damage to the myocardium, encompassing both structural and functional aspects. This mitigation was achieved through the effective inhibition of ferroptosis and oxidative stress. We provide compelling evidence that Taxifolin upregulates the expression of miR-200a, a regulator with a substantial influence on ferroptosis, by activating the Nrf2 signaling pathway.

## 2 Materials and methods

### 2.1 Animals

All animal procedures abided by the National Institutes of Health (USA) guidelines. The animal study was reviewed and approved by Animal Care and Use Committee at the Wenzhou Medical University (wydw2023-0083). At Wenzhou Medical University's Animal Center, we purchased male C57BL/6 mice that were 6–8 weeks old from Zhejiang Vital River Laboratory. Mice were housed in an alternating day/night cycle of 12/12 h, with a temperature of 24 ± 3 °C, an ambient humidity of 50 ± 3 %, and free access to food and water. After euthanasia via pentobarbital overdose, the heart was excised. Tibial length was measured after exposing the tibia by removing the skeletal muscle and soft tissue.

### 2.2 Experimental design

The mice were randomly divided into four groups, each containing 10 mice: (1) Control group, where each mouse was administered with 200 μL/d saline through intragastric (ig) administration for two weeks. A week after the first use of saline, each mouse was administered with 200 μL saline through intraperitoneal (ip) injection; (2) TAX group, where each mouse was administered with TAX through intragastric administration at a dose of 20 mg/kg/d for two weeks. A week after the first use of TAX, each mouse was administered with 200 μL saline through intraperitoneal injection; (3) Dox group, where each mouse was administered with 200 μL/d saline through intragastric administration for two weeks. A week after the first use of saline, each mouse was administered with a 10 mg/kg dose of Dox through intraperitoneal injection. A week after the Dox treatment, mice were weighed and their cardiac functions were measured using echocardiography; (4) TAX plus Dox group, where each mouse was administered with TAX through intragastric administration at a dose of 20 mg/kg/d for two weeks. A week after the first use of TAX, each mouse was administered with a 10 mg/kg dose of Dox through intraperitoneal injection.

### 2.3 Echocardiography

Echocardiography was performed under inhalation anesthesia with 2 % isoflurane using an ultrasound machine. From parasternal long axes, echocardiograms were obtained in M-mode. A technician blinded to the treatments measured the left ventricular ejection fraction (LVEF) and leave ventricular fractional shortening (LVFS) of the mice.

### 2.4 Biochemical analysis

According to the instructions provided by the manufacturer of the biochemical kit, we collected mouse serum and cell culture supernatant after the corresponding treatment and extracted the mouse heart protein and primary cardiomyocyte protein. Lactate dehydrogenase (LDH) (#A020-2-2, Nanjing Jiancheng), creatine kinase MB isoenzyme (CK-MB) (#E006-1-1, Nanjing Jiancheng) assays were performed, Alanine Aminotransferase (ALT) (#C009-2-1, Nanjing Jiancheng), aspartate aminotransferase (AST) (#C010-2-1, Nanjing Jiancheng), blood urea nitrogen (BUN) (#C013-2-1, Nanjing Jiancheng), Malondialdehyde (MDA) (#A003-2-1, Nanjing Jiancheng), reduced glutathione (GSH) (#A006-2-1, Nanjing Jiancheng), and superoxide dismutase (SOD) (#A001-2-2, Nanjing Jiancheng) are all available as assay kits, which were obtained from Nanjing Jiancheng. CTnI ELISA kits (#E-ELM0086c, Elabscience) were provided by Elabscience.

### 2.5 Histopathologic assay

After fixation in 10 % formalin and embedding in paraffin, the section was stained with hematoxylin-eosin (HE). The tissue sample is collected from the experimental animal, usually via biopsy or necropsy. The tissue sample is fixed in a solution such as formalin to preserve its cellular structure. The fixed tissue is dehydrated using a series of increasing concentrations of alcohol, and then embedded in paraffin wax. The embedded tissue is sliced into thin sections, typically 5 μm thick, using a microtome. Each heart is sliced with 10 sections. The sections are mounted on glass slides and stained using various dyes and reagents to highlight different cellular components or structures. The stained sections are examined under a microscope by a trained pathologist or histologist, who identifies any abnormal cellular changes or other pathological features. A light microscope (Olympus BX51) was used to view the stained sections. Some of sections were enlarged by 40 times after HE staining to observe the left and right ventricles. The other sections were enlarged by 400 times to see the inflammatory infiltration in the left ventricle.

### 2.6 Cell culture

H9c2 was obtained from the American Type Culture Collection (ATCC; cat. No: CRL-1446). Cells were cultured at 37 °C in Dulbecco's modified Eagle's medium (DMEM) with 10 % fetal bovine serum (FBS) in a humidified atmosphere.

H9c2 cells were pretreated with 10 μM TAX for 12 h, and then co-treated with 1 μM Dox for 24 h.

### 2.7 Cell viability assay

Prepare the cells: Seed the cells in a 96-well plate or other suitable format and culture them under appropriate conditions until they reach the desired level of confluence or time point. Add the CCK-8 reagent: Remove the medium from the cells and add the CCK-8 reagent to each well. Incubate the plate at 37 °C for a specified period (usually 1 h). Measure absorbance: After the incubation period, measure the absorbance of the formazan dye at 450 nm using a microplate reader. The amount of formazan produced is proportional to the number of viable cells in the culture.

### 2.8 Iron assay

The iron assay kit (ab83366; Abcam) was used to determine the intracellular ferrous iron level. Sample preparation reagents: These reagents are used to prepare the sample for analysis, which may include removing interfering substances and diluting the sample to the appropriate concentration. Iron assay reagents: These reagents typically include a chromogenic substrate that reacts with iron in the sample to produce a color change, which can be measured using a spectrophotometer or colorimeter. Calibration standards: These are solutions of known iron concentrations that are used to create a standard curve and calibrate the assay. Instruction manual: This provides step-by-step instructions for performing the assay, including details on the recommended sample volume, incubation times, and wavelength for measuring the color change. In vivo, after the mice completed the ultrasonic examination, the hearts were excised for iron assay. In vitro, after 24 h of Dox treatment, H9c2 cells could be scraped off and used for iron assay.

### 2.9 ROS/DHE staining assay

The supernatant was removed and rinsed twice in sterile PBS after the treatment. Cells were treated with ROS/DHE (Beyotime, China) working solution, then incubated for 30 min according to manufacturer's instructions. Prepare the cells: Grow the cells under appropriate conditions and treat them as desired. Prepare the staining solution: Dissolve ROS/DHE in dimethyl sulfoxide (DMSO) at a concentration of 10 mM. Prepare a working solution of ROS/DHE by diluting the ROS/DHE stock solution in phosphate-buffered saline (PBS) to a final concentration of 10 μM. Stain the cells: Remove the culture medium from the cells and replace it with fresh medium containing the DHE working solution. Incubate the cells for 30 min to 1 h at 37 °C in the dark. Image the cells: After staining, wash the cells with PBS to remove excess dye. Visualize the cells using a fluorescence microscope or flow cytometer with excitation at 488 nm and emission at 590 nm.

### 2.10 Cellular immunofluorescence

Immunofluorescence staining is performed in H9c2. Fixing the cells with 4 % paraformaldehyde for 10 min, Interrupting the membrane with 0.3 % Triton for 10 min, blocking with 5 % BSA for 2 h, and incubating overnight at 4 °C with the corresponding primary antibody, the membrane is fixed. Incubate the secondary antibody for 1 h in the dark, followed by the DAPI. After the procedure has been completed, we take photographs using an anti-fluorescence quenching solution.

### 2.11 Transient transfection of siRNA

The sense sequence for the Nrf2 small RNA interference (siRNA) was 5′- GCCTTACTCTCCCAGTGAATA -3'. The siRNA was transfected into H9c2 cells using Lipofectamine TM 3000 reagent (Invitrogen, Carlsbad). Prepare the siRNA: The siRNA of interest is prepared in a suitable buffer, such as Opti-MEM. Dilute the Lipofectamine™ 3000 reagent: The Lipofectamine™ 3000 reagent is diluted in Opti-MEM to the appropriate concentration mix the reagents: The diluted Lipofectamine™ 3000 reagent is mixed with the DNA or RNA of interest, and incubated for a period of time to allow the formation of the liposomes. Add the mixture to cells: The mixture of Lipofectamine™ 3000 reagent and DNA or RNA is added to cells in culture and incubated for a specified period of time. Evaluate transfection efficiency: The cells are evaluated for transfection efficiency, which may be assessed using various techniques such as fluorescence microscopy, flow cytometry, or western blotting.

### 2.12 Real-time quantitative PCR

The total RNA was extracted from cells using the TRIzol reagent (Invitrogen, Carlsbad, CA). The ReverTra Ace QPCR RT Kit was used to reverse-transcribe RNA (Toyobo, Shanghai). IQ SYBR Green Supermix (BioRad, Hercules, CA) was used for RT-PCR. The housekeeping gene was GAPDH. With the Bulge-Loop miRNA qRT-PCR Starter Kit (Ribobio Technology, Guangzhou), miRNA levels were determined. The internal control of miRNA was U6.

### 2.13 Inhibitor-miR transfection

The normal control and inhibitor of miR-200a was constructed by Guangzhou Ribobio Co Ltd and transfected with Lipofectamine 3000 (Invitrogen, Carlsbad) for 6 h, then cultured 48 h to collect H9c2 cells.

### 2.14 Western blotting analysis

Extract the protein from the sample of interest and quantitate the amount of protein using a protein assay. Using the BCA kit (#P0012, Beyotime), protein concentration was determined. Load the protein sample onto a polyacrylamide gel and run the gel using an electric field to separate the proteins based on their size. The proteins are then transferred from the gel to a membrane using a transfer apparatus, such as a semi-dry or wet transfer system.20–40 mg of protein was loaded for SDS-PAGE before PVDF membrane (#ISEQ85R, Millipore Corporation) transfer. Membranes were blocked by 5 % skim milk for 3 h and then incubated with primary antibody overnight at 4 °C. After 16 h, membranes incubated with goat anti-rabbit IgG, peroxidase conjugated (#bl003a, Biosharp) for 3 h, then washed twice. The target protein signal was visualized using a ChemiDoc™ XRS + system and Image Lab software (Bio-Rad). The protein signal intensity was detected using Image J. We evaluated PTGS2, GPX4, Nrf2, HO-1 and NQO1 levels using Western blotting analysis.

### 2.15 Reagents and antibodies

**Fig. 1:**
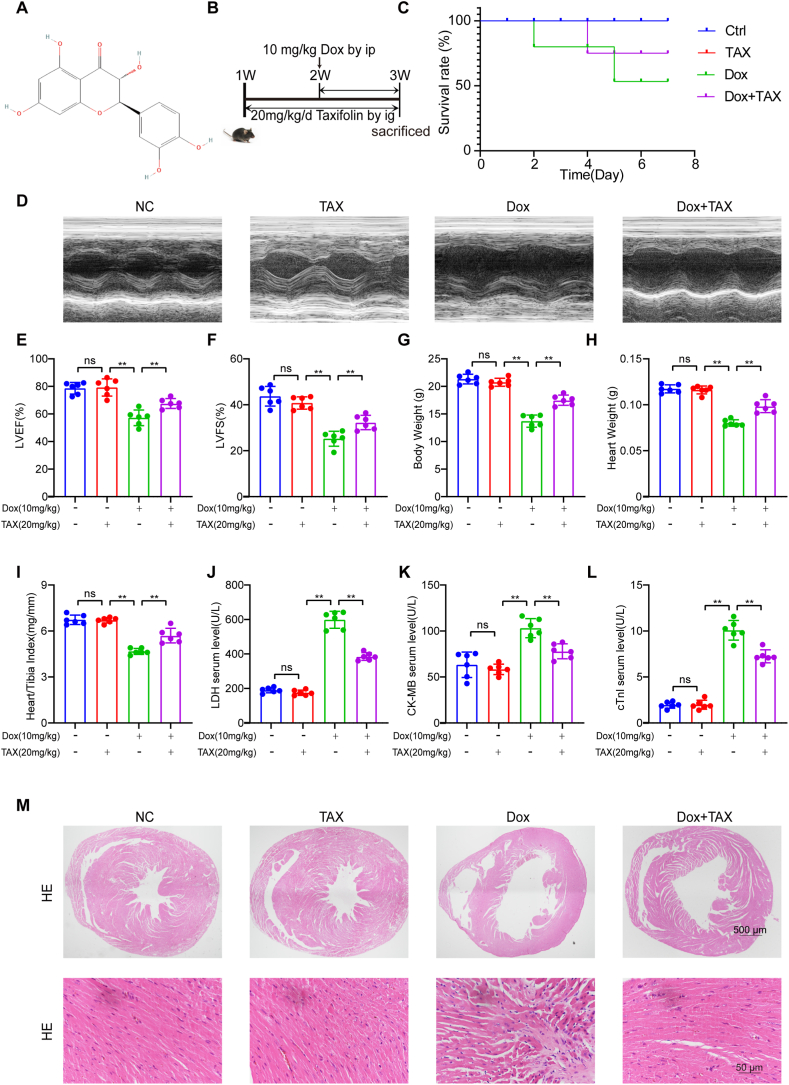
Abbreviations: CK-MB, creatine kinase MB isoenzyme; Doxorubicin, Dox; Hematoxylin-eosin, HE; LDH, lactate dehydrogenase; LVEF, left ventricular ejection fraction; LVFS, leave ventricular fractional shortening; NC, Normal Control; TAX, taxifolin. TAX attenuated Dox-induced cardiotoxicity in vivo. (A) Chemical structure of Taxifolin (B) Schematic protocol for mice treatments (C) Kaplan-Meier survival curves of mice (D) Representative images of each group's echocardiogram. (E–F) LVEF and LVFS, n = 6. (G–I) Body weight, heart weight and heart/Tibia index, n = 6 (J–L) LDH, CK- MB, and cTnI levels in mouse serum, n = 6. (M) Representative images of HE staining, original magnification, ×40, scale bar, 500 μm; original magnification, ×400, scale bar, 50 μm. n = 6. Data are means ± SD, **P < 0.01.

Taxifolin (C_15_H_12_O_7_, purity ≥99 %, Fig. 1A) was bought from MCE. Doxorubicin (#D1515, Sigma-Aldrich) was purchased from Sigma-Aldrich. The primary antibodies used were GPX4 (#A11243, ABclonal), PTGS2 (#A1253, ABclonal), P62 (#23214, CST), Nrf2 (#12721, CST), NQO1 (#11451-1-AP, Proteintech), HO-1 (#10701-1-AP, Proteintech), β-actin (#AC026, ABclonal).

### 2.16 Statistical analysis

We analyzed the data using SPSS version 22.0 and presented the results as mean ± SEM. The data we collected were all normally distributed. The two experimental groups were compared using the unpaired Student's t-test. A one-way analysis of variance followed by Duncan's T3 multiple-range test was used for comparisons of more than two groups. Statistical significance was determined by *P*-values less than 0.05.

## 3 Results

### 3.1 TAX attenuated dox-induced cardiotoxicity in vivo

The chemical structure of TAX was shown in Fig. 1A. In a preliminary experiment, we evaluated the effects of different concentrations of TAX (10, 20, 40 mg/kg/d) on Dox-induced cardiotoxicity and measured several parameters including LDH, CK-MB, and cTnI (Supplemental Figs. 1A–C). We found that the optimal concentration of TAX in vivo was 20 mg/kg, based on these measurements. Therefore, we used this concentration for subsequent in vivo experiments. The study utilized mice to create a model of cardiotoxicity through the administration of Dox (Fig. 1 B). The mortality was 50 % in the Dox group, but only 70 % in the TAX plus Dox group (Fig. 1C). Echocardiography was conducted for each group, and the Dox group had notably lower LVEF (57.19 ± 5.68) and LVFS (25.21 ± 3.25) values than the control group, while the TAX plus Dox groups had significantly higher values in LVEF (67.63 ± 3.56) and LVFS (32.3 ± 3.13) (Fig. 1D, E, F). Furthermore, Dox-induced cardiotoxicity caused a decrease in body weight, heart weight, and heart weight/tibial length, which were alleviated by TAX (Fig. 1 G, H, I). LDH levels (598.3 ± 49.29) were increased in Dox group, while TAX could effectively reduce LDH levels (385.3 ± 22.05) (Fig. 1J). Similar expressions levels of CK-MB (103 ± 10.38) and cTnI (10.08 ± 1.07) were also elevated in Dox group, while TAX could effectively reduce the expressions of CK-MB (77.97 ± 8.15) and cTnI (7.25 ± 0.71) (Fig. 1K and L). HE staining also revealed that Dox-treated mice showed Left ventricle dilatation, which was reversed by TAX. TAX prevented histopathological damage to the cardiac tissues of mice caused by Dox (Fig. 1M). In addition, we find that TAX could reduce Dox-induced hepatotoxicity, nephrology (Supplemental Figs. 1D–F).

### 3.2 TAX attenuated dox-induced ferroptosis in vivo

**Fig. 2:**
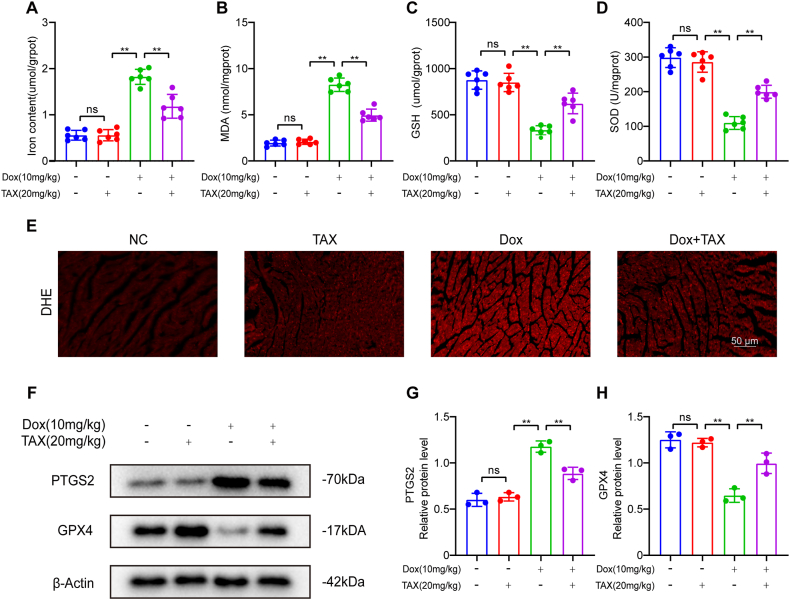
TAX attenuated Dox-induced ferroptosis in vivo. Abbreviations: DHE, Dihydroethidium; Doxorubicin, Dox; GPX4, Glutathione peroxidase 4; GSH, reduced glutathione; MDA, malondialdehyde; NC, Normal Control; PTGS2, Prostaglandin-endoperoxide synthase 2; SOD, superoxide dismutase; TAX, taxifolin. (A–D) Fe^2+^, MDA, GSH and SOD in vivo, n = 6 (E) Oxidative stress indicator staining, red indicates DHE, original magnification, ×400, scale bar, 50 μm, n = 3. (F) PTGS2 and GPX4 protein expression as assayed by Western blotting in vivo. Unprocessed images of Western blotting were in the supplementary material 1. (G–H) Quantitative analysis of the expression of PTGS2 and GPX4 proteins. The expression levels were normalized to β-actin, n = 3. Data are means ± SD, **P < 0.01. (For interpretation of the references to color in this figure legend, the reader is referred to the Web version of this article.)

To investigate the effects of TAX on Dox-induced ferroptosis in mice, we measured levels of ferroptosis and oxidative stress after treatment with TAX. The results were clear: in cardiac tissue, Dox increased ferrous iron levels (1.82 ± 0.16), while TAX could inhibit ferrous iron levels (1.18 ± 0.26) (Fig. 2A). MDA, a byproduct associated with lipid peroxidation, was increasing (8.26 ± 0.72) in Dox groups, but TAX effectively inhibited MDA levels (4.97 ± 0.25) in Dox model (Fig. 2B). As for anti-ferroptosis markers GSH (332.5 ± 49.78) and SOD (109.7 ± 18.49), we found that they were significantly reduced in the Dox groups, but TAX effectively reversed GSH (622.9 ± 111.3) and SOD levels (199.9 ± 18.66) (Fig. 2C and D). Moreover, while Dox increased DHE in cardiac tissue, TAX decreased it (Fig. 2E). To better assess the changes in ferroptosis levels in myocardial tissue, we used Western blotting to detect changes in proteins related to the disease (Fig. 2F). As a result of Dox-induced cardiotoxicity, PTGS2 expression increased and GPX4 expression decreased (Fig. 2G and H).

### 3.3 TAX attenuated dox-induced cardiotoxicity and ferroptosis in vitro

**Fig. 3:**
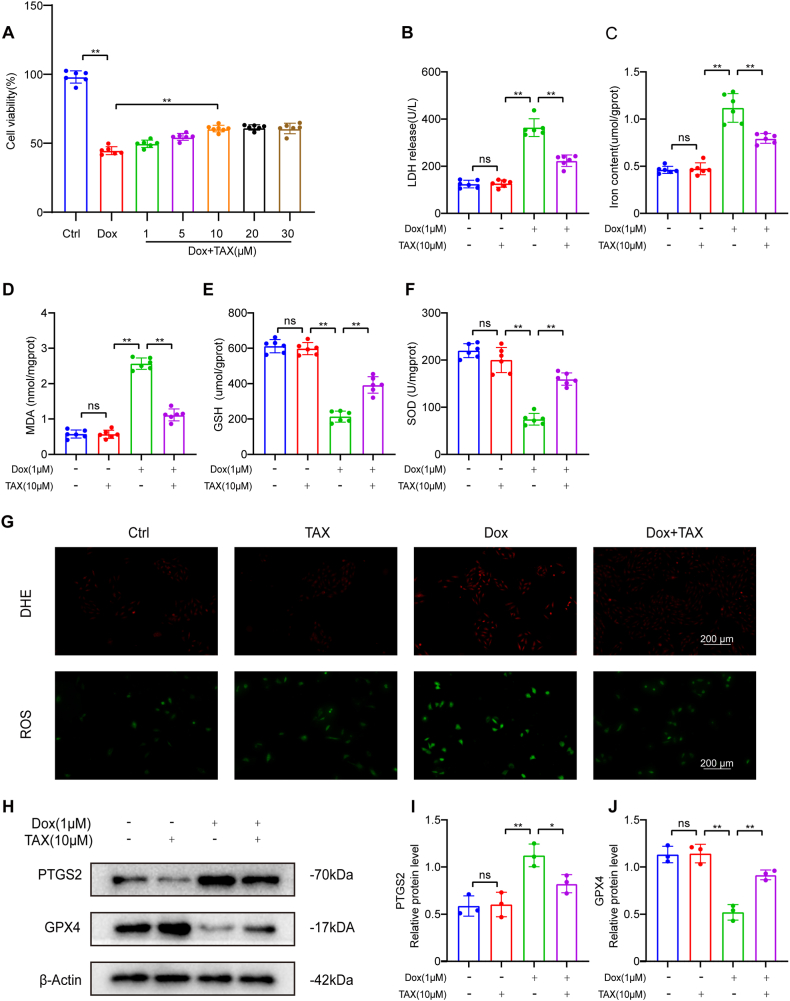
TAX attenuated Dox-induced ferroptosis in vitro. Abbreviations: Ctrl, Control; DHE, Dihydroethidium; Doxorubicin, Dox; GPX4, Glutathione peroxidase 4; GSH, reduced glutathione; LDH, lactate dehydrogenase; MDA, malondialdehyde; PTGS2, Prostaglandin-endoperoxide synthase 2; ROS, Reactive Oxygen Species; SOD, superoxide dismutase; TAX, taxifolin. (A) Cell viability assay in each group, n = 6. (B) LDH, n = 6. (C–F) Fe^2+^, MDA, GSH and SOD in H9c2 cells, n = 6 (G) Oxidative stress indicator staining, green indicates ROS and red indicates DHE, original magnification, ×100, scale bar, 200 μm, n = 3. (H) PTGS2 and GPX4 protein expression as assayed by Western blotting in vitro. Unprocessed images of Western blotting were in the supplementary material 1. (I–J) Quantitative analysis of the expression of PTGS2 and GPX4 proteins. The expression levels were normalized to β-actin, n = 3. Data are means ± SD, **P < 0.01. (For interpretation of the references to color in this figure legend, the reader is referred to the Web version of this article.)

The results showed that TAX treatment significantly improved Dox-induced injury to H9c2 cells. The optimal concentration of TAX, as determined by CCK8 assay, was found to be 10 M (Fig. 3A). Treatment with TAX resulted in a reduction in LDH levels in the supernatant of the cells (Fig. 3B). The effects of TAX on Dox-induced ferroptosis in H9c2 cells were then studied, and changes in ferroptosis and oxidative stress were observed after treatment. Specifically, TAX significantly reduced ferrous iron and MDA levels (Fig. 3C and D) while promoting GSH and SOD production (Fig. 3E and F). Additionally, by detecting ROS and DHE [37,38], we found that TAX inhibited ROS and DHE production (Fig. 3G). To better assess the changes in ferroptosis levels, Western blotting was used to detect changes in ferroptosis-related proteins in H9c2 cells (Fig. 3H). As a result of Dox toxicity, PTGS2 expression increased and GPX4 expression decreased in H9c2 cells, but these changes were alleviated by TAX (Fig. 3I and J). Overall, these findings suggest that TAX effectively alleviates ferroptosis and protects cardiomyocytes.

### 3.4 TAX adjusted Nrf2 signaling pathway in vivo and in vitro

**Fig. 4:**
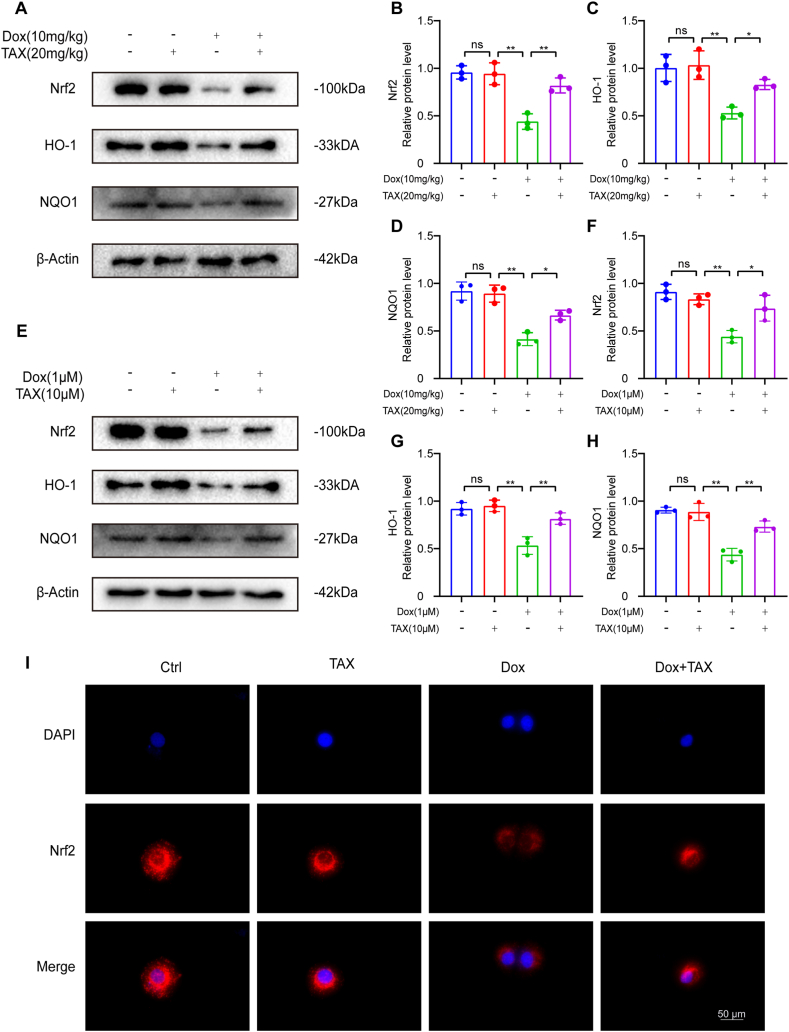
TAX activated Nrf2 signal pathway in vivo and in vitro. Abbreviations: Ctrl, Control; Doxorubicin, Dox; GPX4, Glutathione peroxidase 4; HO-1, Heme Oxygenase-1; NQO1, NADPH: Quinone Oxidoreductase 1; Nrf2, Nuclear factor erythroid 2-related factor 2; TAX, taxifolin. (A) Nrf2, HO-1 and NQO1 protein expression as assayed by Western blotting in vivo. Unprocessed images of Western blotting were in the supplementary material 1. (B–D) Quantitative analysis of the expression of Nrf2, HO-1 and NQO1 proteins. The expression levels were normalized to β-actin, n = 3. (E) Nrf2, HO-1 and NQO1 protein expression as assayed by Western blotting in vitro. Unprocessed images of Western blotting were in the supplementary material 1. (F–H) Quantitative analysis of the expression of Nrf2, HO-1 and NQO1 proteins. The expression levels were normalized to β-actin, n = 3. (I) Immunofluorescence staining of Nrf2 in each group, original magnification, ×400, scale bar, 50 μm, n = 3. Data are means ± SD, *P < 0.05; **P < 0.01.

Nrf2 signaling pathway is associated with ferroptosis [39]. In myocardial tissue treated with TAX, we observed changes in Nrf2 signaling following ferroptosis by Western blotting (Fig. 4A). Dox treatment significantly reduced Nrf2 and downstream proteins HO-1 and NQO1 in mouse heart tissues. However, TAX treatment significantly increased the levels of Nrf2, HO-1, and NQO1 compared to the model group (Fig. 4B, C, D). Western blotting assays were also used to assess Nrf2, HO-1, and NQO1 expression in H9c2 cells (Fig. 4E). In vitro, Dox significantly decreased Nrf2, HO-1, and NQO1 protein expression levels, while TAX increased them (Fig. 4F, G, and H). Immunofluorescence microscopy of H9c2 cells stained for Nrf2 also showed the same results: Nrf2 expression was increased by TAX (Fig. 4I). Based on these findings, TAX increased Nrf2 expression and subsequently affected HO-1 and NQO1 expression levels.

### 3.5 TAX attenuated doxorubicin-induced cardiotoxicity and ferroptosis via activation of Nrf2

**Fig. 5:**
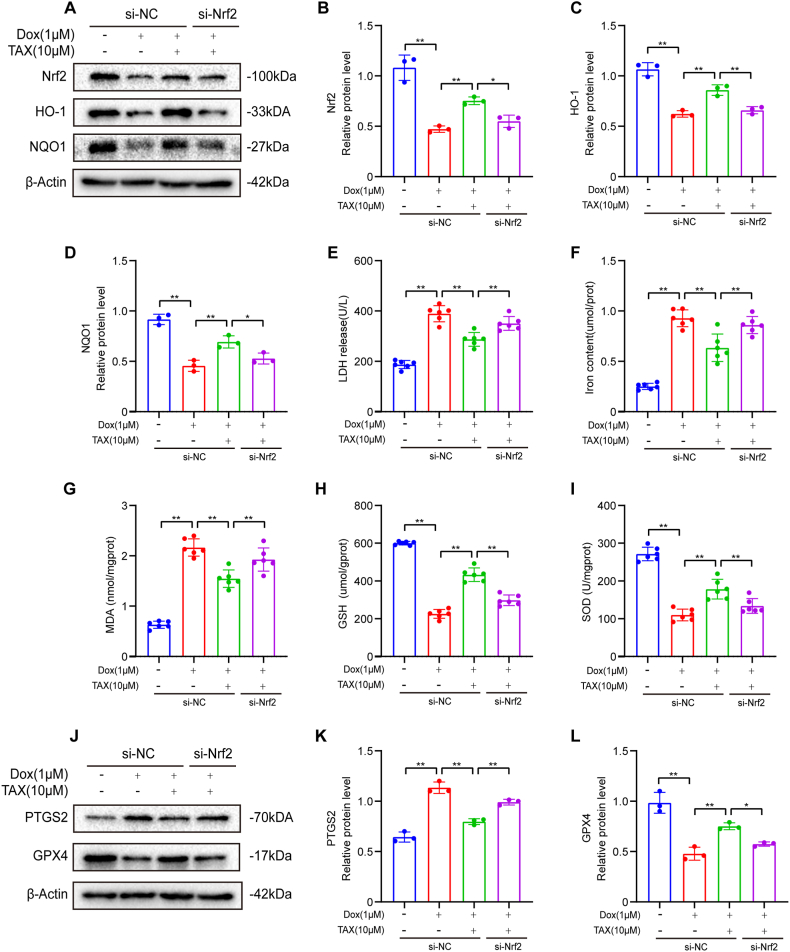
TAX attenuated doxorubicin-induced ferroptosis via Nrf2 signal pathway in vitro. Abbreviations: Doxorubicin, Dox; GPX4, Glutathione peroxidase 4; GSH, reduced glutathione; HO-1, Heme Oxygenase-1; LDH, lactate dehydrogenase; MDA, malondialdehyde; NC, Normal Control; NQO1, NADPH: Quinone Oxidoreductase 1; Nrf2, Nuclear factor erythroid 2-related factor 2; PTGS2, Prostaglandin-endoperoxide synthase 2; SOD, superoxide dismutase; TAX, taxifolin. (A) Nrf2, HO-1 and NQO1 protein expression as assayed by Western blotting in vitro. Unprocessed images of Western blotting were in the supplementary material 1. (B–D) Quantitative analysis of the expression of Nrf2, HO-1 and NQO1 proteins. The expression levels were normalized to β-actin, n = 3. (E) LDH, n = 6. (F–I) Fe^2+^, MDA, GSH and SOD in vitro, n = 6 (J) PTGS2 and GPX4 protein expression as assayed by Western blotting in vitro. Unprocessed images of Western blotting were in the supplementary material 1. (K–L) Quantitative analysis of the expression of PTGS2 and GPX4 proteins. The expression levels were normalized to β-actin, n = 3. Data are means ± SD, *P < 0.05; **P < 0.01.

Nrf2 siRNA was used in H9c2 cells before Dox treatment. With Nrf2 siRNA, Nrf2 protein expression was downregulated in H9c2 cells (Fig. 5A and B) and the protein expression levels of some downstream proteins, such as HO-1 and NQO1, were significantly decreased (Fig. 5A, C, D). The therapeutic effect of TAX was partially reversed by si-Nrf2. With si-Nrf2, LDH, ferrous iron levels, and MDA levels increased, while GSH and SOD levels decreased (Fig. 5E, F, G, H, I). By using immuno-blotting, we found that si-Nrf2 treatment increased PTGS2 expression (Fig. 5J and K) and reduced GPX4 expression after Dox-induced injury, which were the exact opposite of the results observed with TAX treatment.

### 3.6 TAX adjusted Nrf2 signaling pathway and attenuated ferroptosis by miR-200a

**Fig. 6:**
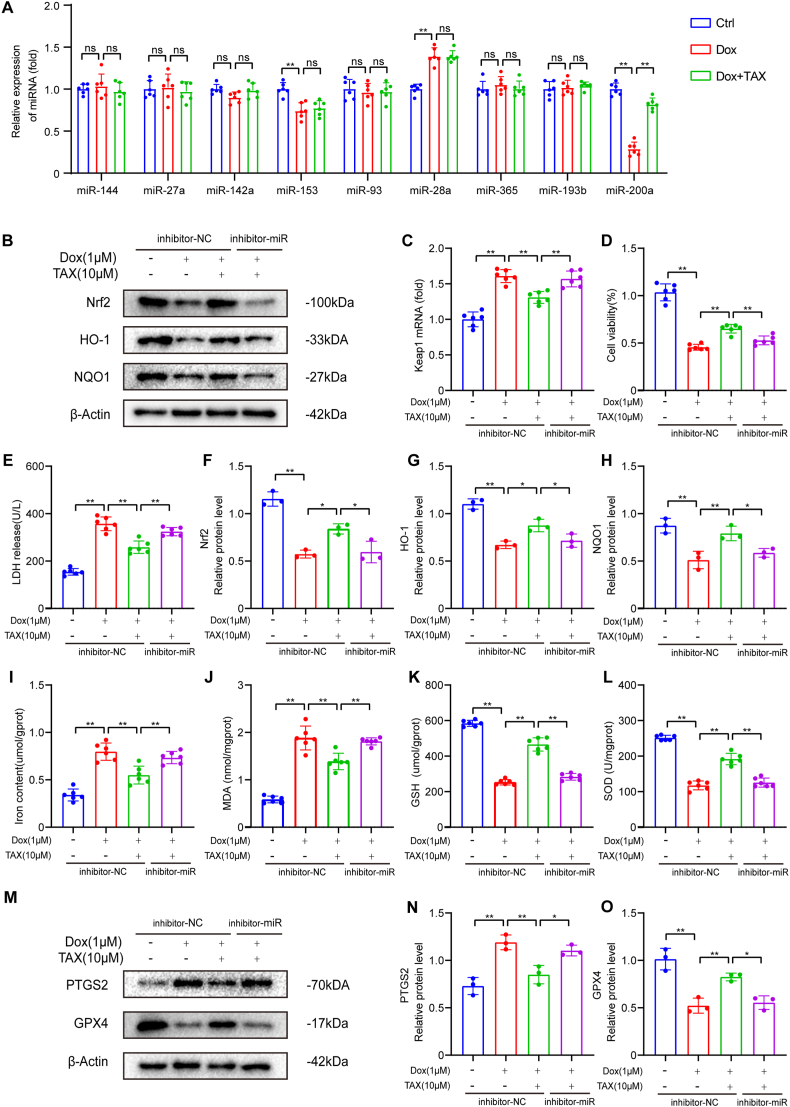
TAX adjusted Nrf2 signal pathway and attenuated ferroptosis by miR-200a. Abbreviations: Doxorubicin, Dox; GPX4, Glutathione peroxidase 4; GSH, reduced glutathione; HO-1, Heme Oxygenase-1; Keap1, Kelch-like ECH-associated protein 1; LDH, lactate dehydrogenase; MDA, malondialdehyde; NC, Normal Control; NQO1, NADPH: Quinone Oxidoreductase 1; Nrf2, Nuclear factor erythroid 2-related factor 2; PTGS2, Prostaglandin-endoperoxide synthase 2; SOD, superoxide dismutase; TAX, taxifolin. (A) The expression levels of Nrf2-related miRNA in H9c2 cells. (B) Nrf2, HO-1 and NQO1 protein expression as assayed by Western blotting in vitro. Unprocessed images of Western blotting were in the supplementary material 1. (C) The expression levels of Keap1 mRNA, n = 6. (D) Cell viability assay in each group, n = 6. (E) LDH, n = 6. (F–H) Quantitative analysis of the expression of Nrf2, HO-1 and NQO1 proteins. The expression levels were normalized to β-actin, n = 3. (I–L) Fe^2+^, MDA, GSH and SOD in vitro, n = 6 (M) PTGS2 and GPX4 protein expression as assayed by Western blotting in vitro. Unprocessed images of Western blotting were in the supplementary material 1. (N–O) Quantitative analysis of the expression of PTGS2 and GPX4 proteins. The expression levels were normalized to β-actin, n = 3. Data are means ± SD, *P < 0.05; **P < 0.01.

In the following experiments, we determined which miRNAs affect Nrf2 transcription in H9c2 cells after Dox and TAX treatment. It was found that all the miRNAs known to regulate Nrf2 level were detected, including miR-144, miR-27a, miR-142a, miR-153, miR-93, miR-28a, miR-365, miR-193b and miR-200a [30]. As a result of DOX treatment, the levels of miR-28a were upregulated, but those of miR-153 and miR-200a were decreased. At the same time, we found that TAX only affected the expression of miR-200a (Fig. 6 A). Therefore, we suggest that TAX may be involved in Nrf2 activation and ferroptosis by influencing miR-200a. MiR-200a activated Nrf2 by targeting Keap1 mRNA and promoting its degradation. To prove our conjecture, we used an inhibitor of microRNA to suppress miR-200a. We assessed the expression level of Keap1 mRNA and showed that they were increased after TAX treatment, while inhibitor-miR decreased the expression level of Keap1 mRNA (Fig. 6 C). As we expected, inhibitor-miR reversed the therapeutic effect of TAX and increased cytotoxicity and LDH level (Fig. 6 D, E). To investigate the role of miR-200 in TAX-mediated Nrf2 activation, we measured the Nrf2 protein expression and found that inhibitor-miR decreased the Nrf2 protein expression by using Western blotting analysis (Fig. 6 B, F). Meanwhile, the expression of downstream proteins HO-1 and NQO1 were also decreased with inhibitor-miR (Fig. 6 B, G, H). Additionally, ferrous iron and MDA levels increased (Fig. 6 I, J), while GSH and SOD levels decreased with inhibitor-miR (Fig. 6 K, L). Furthermore, inhibitor-miR increased PTGS2 expression and decreased GPX4 expression after Dox-induced injury, which was opposite to the effects of TAX (Fig. 6 M, N, O). These findings suggest that the miR-200a is involved in TAX-mediated Nrf2 activation and subsequent protection against Dox-induced injury in H9c2 cells.

## 4 Discussion

**Fig. 7:**
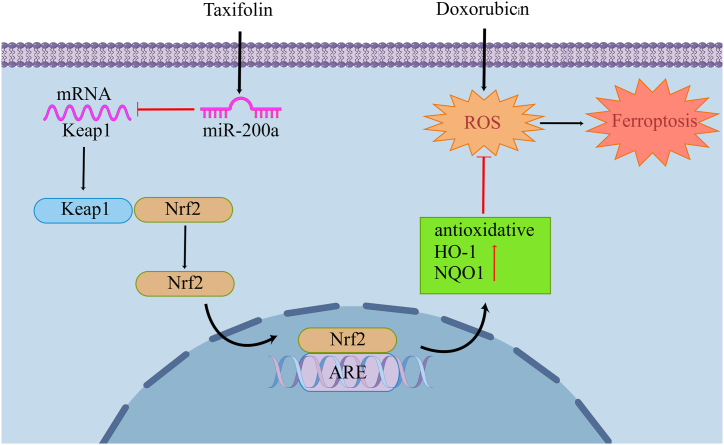
Schematic: Taxifolin protects against doxorubicin-induced cardiotoxicity and ferroptosis by adjusting microRNA-200a-mediated Nrf2 signaling.

TAX treatment was found to improve Doxorubicin-induced injury in H9c2 cells and in mouse heart tissues (Fig. 7). The therapeutic effect of TAX was attributed to its ability to inhibit ferroptosis and reduce oxidative stress levels. Additionally, TAX was found to regulate the Nrf2 signaling pathway, leading to increased expression of Nrf2 and downstream proteins such as HO-1 and NQO1. Inhibition of this pathway by si-Nrf2 or inhibitor-miR partially reversed the protective effect of TAX. Overall, these findings suggest that TAX has the potential to attenuate Doxorubicin-induced cardiotoxicity by regulating the miR-200a-Keap1-Nrf2 signaling pathway and inhibiting ferroptosis and oxidative stress.

Dox-induced cardiotoxicity refers to the damage or injury that the chemotherapy drug Dox can cause to the heart muscle [40]. Dox is a widely used chemotherapy drug for treating various types of cancer, but it can have negative effects on the heart, leading to heart failure and other cardiac problems [41]. The exact mechanism of Dox-induced cardiotoxicity is not completely understood, but it is believed to be related to the drug's ability to generate ROS and induce oxidative stress, which can damage the heart muscle cells [42]. Apoptosis and oxidative stress contribute to the cardiotoxicity caused by Dox [43,44]. Cardiac fibrosis is a common feature in the evolution of various cardiovascular diseases, and Dox eventually leads to myocardial fibrosis [[45], [46], [47], [48]]. This damage can lead to a reduction in the heart's ability to pump blood effectively and may cause symptoms such as shortness of breath, fatigue, and edema [49]. Preventing or reducing Dox-induced cardiotoxicity is an important consideration in cancer treatment, and various strategies have been explored to mitigate its effects.

Ferroptosis is a type of programmed cell death that is characterized by the accumulation of lipid peroxides in the cell membrane, leading to oxidative damage and cell death [50]. It has been proposed to be involved in the pathogenesis of various diseases, including cancer, neurodegenerative diseases, and cardiovascular diseases [51,52]. Doxorubicin-induced cardiotoxicity is a well-known side effect of doxorubicin, a widely used chemotherapeutic drug for the treatment of various cancers. Doxorubicin-induced cardiotoxicity is characterized by the ROS, lipid peroxidation, and mitochondrial dysfunction, which can lead to cardiomyopathy [53]. Recent studies have suggested that ferroptosis may play a role in doxorubicin-induced cardiotoxicity [54]. In particular, doxorubicin has been shown to induce lipid peroxidation and ferroptosis in cardiomyocytes, and inhibition of ferroptosis has been shown to attenuate doxorubicin-induced cardiotoxicity in animal models [55]. Therefore, the regulation of ferroptosis may be a promising strategy for the prevention and treatment of doxorubicin-induced cardiotoxicity.

TAX, a potential ferroptosis inhibitor, has been shown to protect cardiomyocytes from doxorubicin-induced injury by inhibiting ferroptosis and oxidative stress. TAX inhibits ferroptosis in cardiomyocytes and promotes GPX4 expressing. A key target for ferroptosis is GPX4, which prevents lipid peroxidation and maintains lipid bilayer homeostasis. After TAX treatment, left ventricular dilation and inflammatory infiltration induced by Dox were significantly improved.

Nrf2 signaling pathway plays an important role in regulating cellular responses to oxidative stress and maintaining cellular redox balance [56]. Under normal conditions, the protein Keap1 interacts with Nrf2 and promotes its ubiquitination and degradation, preventing its translocation to the nucleus and activation of downstream antioxidant response genes [57]. However, under conditions of oxidative stress, the protein p62/SQSTM1 can bind to Keap1 and disrupt its interaction with Nrf2, allowing Nrf2 to translocate to the nucleus and activate the transcription of antioxidant and detoxification genes [58]. Compared to the control group, Nrf2 expression was significantly reduced in the hearts of the Dox group. It may be due to the inhibition of Nrf2 that Dox mice develop cardiac iron toxicity. As a result of TAX treatment, Nrf2 related pathways were activated and iron toxicity was inhibited. After Nrf2 siRNA treatment, the effect was lost, suggesting that TAX modulates Nrf2 signaling to blunt iron atrophy in Dox-treated mice. MiR-200a has been reported to target Nrf2 and inhibit its expression in some studies [59]. For example, one study showed that miR-200a could directly bind to the 3′ untranslated region (UTR) of Nrf2 mRNA and repress its translation, leading to decreased expression of downstream antioxidant enzymes and increased oxidative stress in renal cells. However, other studies have reported conflicting results, with some showing that miR-200a could upregulate Nrf2 expression by targeting other negative regulators of Nrf2. Overall, the regulation of Nrf2 by miR-200a appears to be complex and context-dependent, and more research is needed to fully elucidate the mechanisms involved. The use of TAX to regulate this pathway has shown promising results in attenuating doxorubicin-induced cardiotoxicity. We demonstrated that treatment with Dox can significantly damage cardiomyocytes, in vitro and in vivo, by decreasing miR-200a-Keap1-Nrf2 signaling pathways. In contrast, treatment with TAX increased Nrf2 expression and related pathways through activation of miR-200a-Keap1-Nrf2 signaling pathways, thereby reducing doxorubicin-induced cardiotoxicity and ferroptosis. After treatment with Nrf2 siRNA and inhibitor-miR, the effect of alleviating doxorubicin-induced cardiotoxicity and ferroptosis was lost. According to these results, TAX could attenuate ferroptosis in the heart of Dox mice by modulating the miR-200a-Keap1-Nrf2 signaling pathways. AMPK plays a critical role in controlling inflammation and cardiac homeostasis, and Taxifolin may protects against doxorubicin-induced cardiotoxicity through AMPK [37,48].

In conclusion, the results of this study suggest that TAX has a protective effect against doxorubicin-induced cardiotoxicity in mice, which is attributed to the inhibitor of ferroptosis and oxidative stress. This protective effect is achieved through the regulation of the miR-200a-Keap1-Nrf2 signaling pathway, which increases Nrf2 expression and downstream antioxidant protein expression such as HO-1 and NQO1. These findings provide insights into the mechanisms underlying the protective effects of TAX in doxorubicin-induced cardiotoxicity and suggest that TAX may be a promising therapeutic option for the prevention or treatment of doxorubicin-induced cardiotoxicity in the future.

## Data availability

All data associated with my study was not deposited into a publicly available repository. And all data will be made available on request.

## Funding

None.

## Declaration of competing interest

The authors declare that they have no known competing financial interests or personal relationships that could have appeared to influence the work reported in this paper.
