# Supplementary material for: Taxifolin protects against doxorubicin-induced cardiotoxicity and ferroptosis by adjusting microRNA-200a-mediated Nrf2 signaling pathway
Source: Heliyon. 2023 Nov 8;9(11):e22011. doi: 10.1016/j.heliyon.2023.e22011 (PMC10694176; doi:10.1016/j.heliyon.2023.e22011)

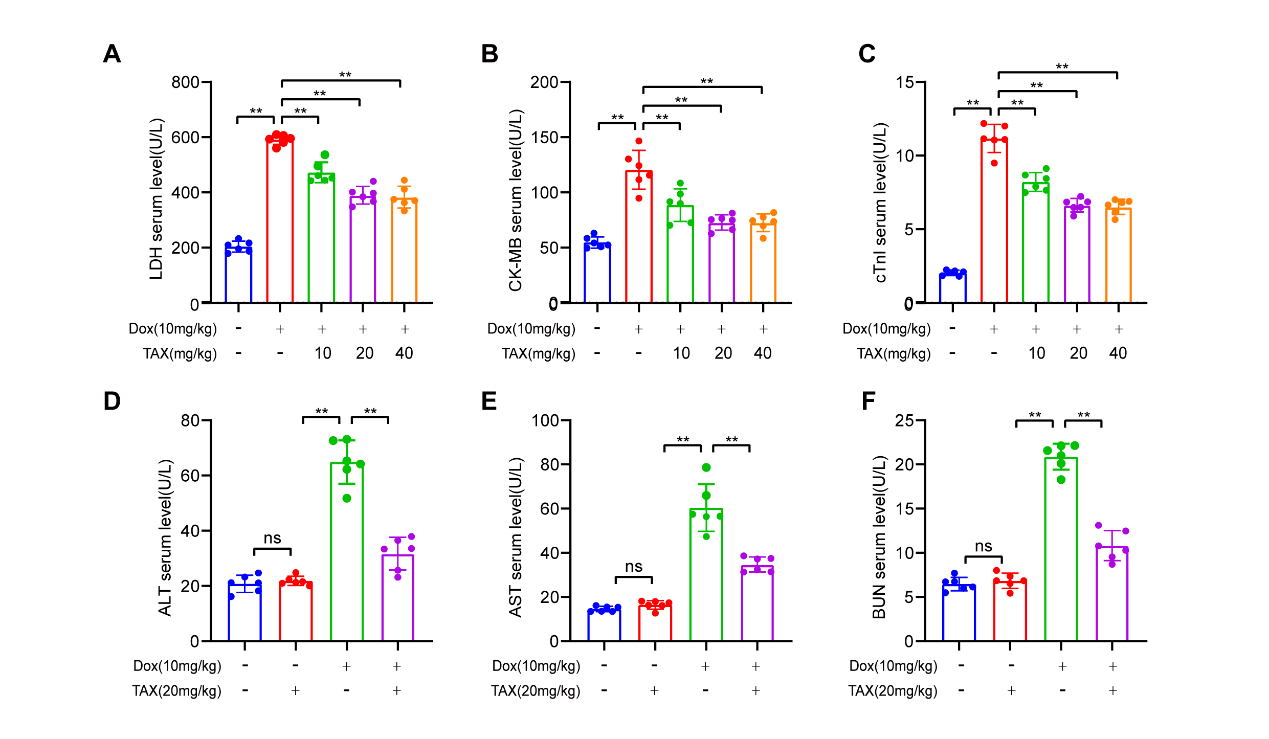


**Supplemental Fig.1 (A-C) LDH, CK- MB, and cTnI levels in mouse serum, n = 6. (D-F) ALT, AST, BUN levels in mouse serum, n = 6.**

**Supplemental Fig.2 Unprocessed images of Western blotting (WB) assays.**

**Figure 2F**

PTGS2:
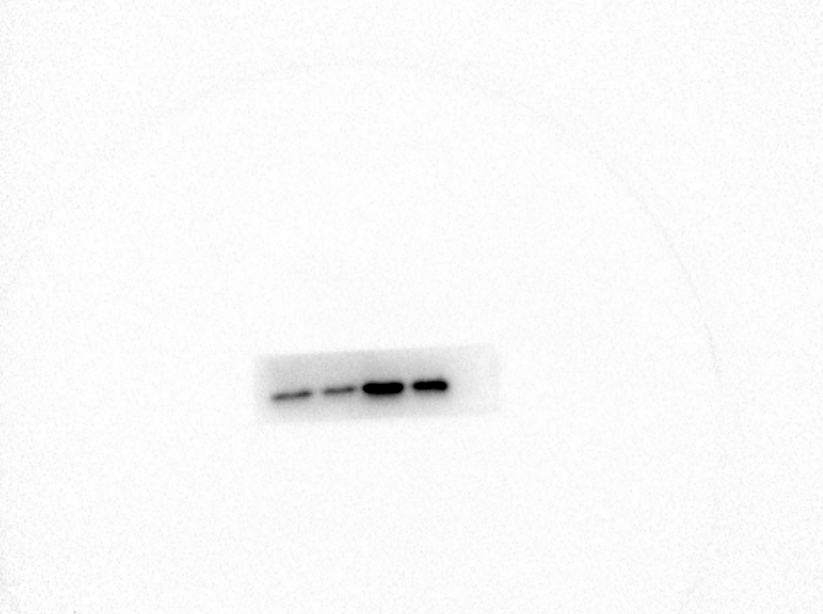


GPX4:
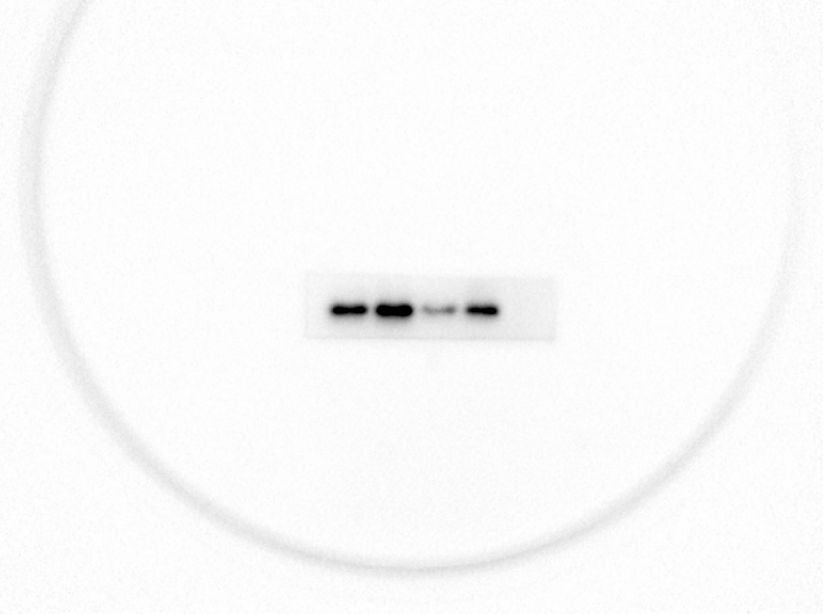


β-Actin:
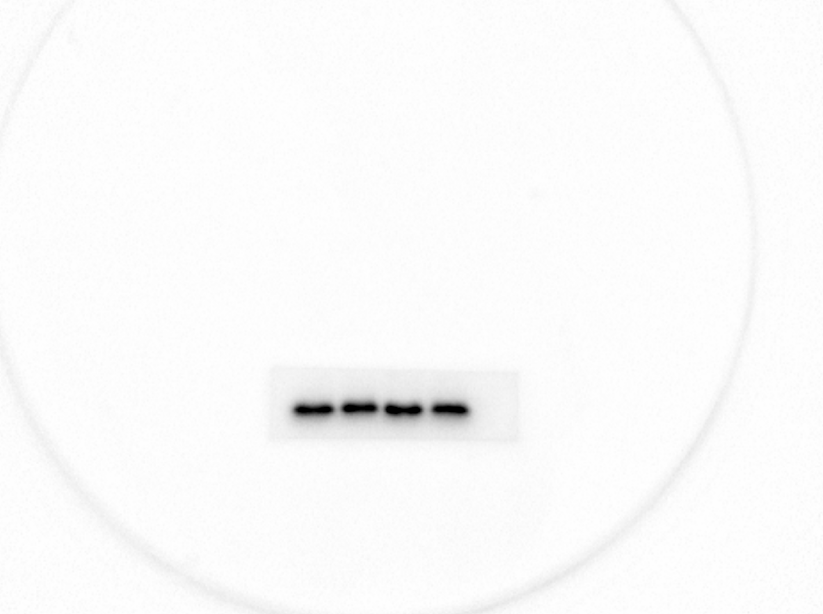


**Figure 3H**

PTGS2:
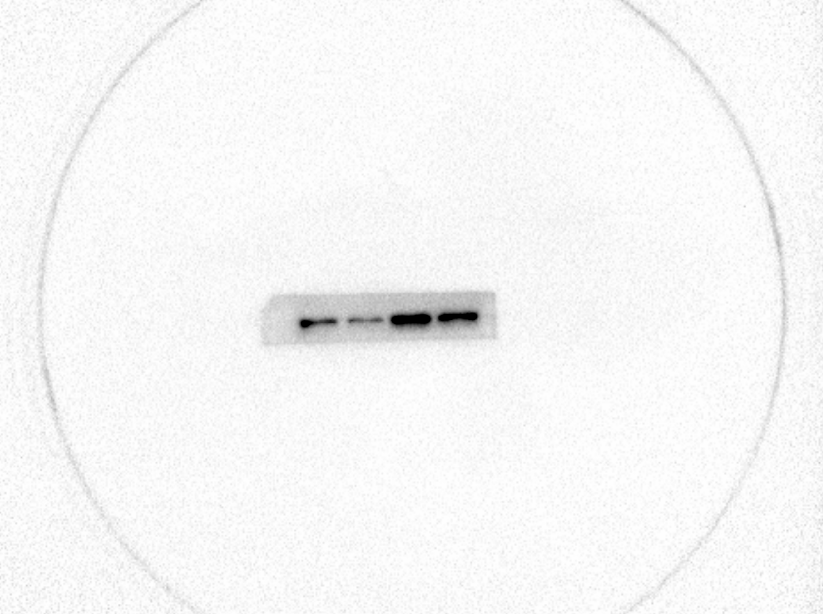


GPX4:
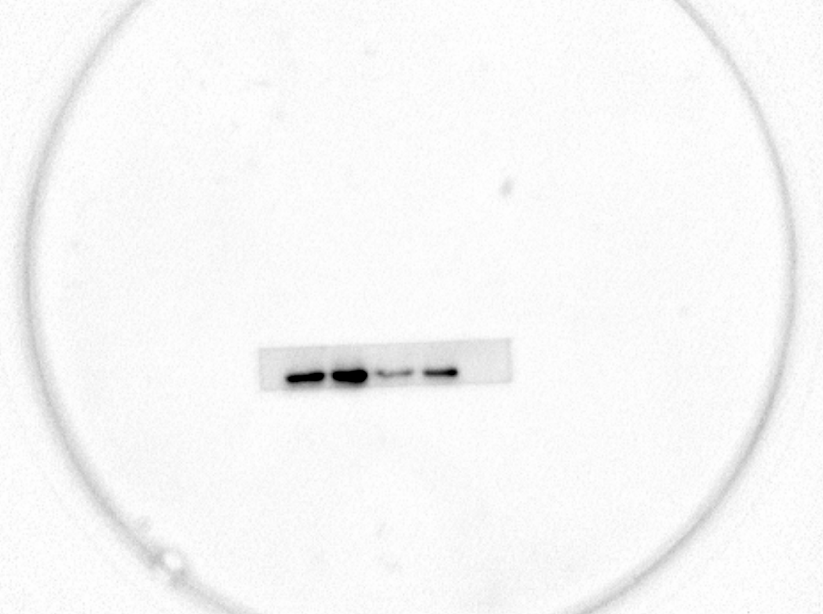


β-Actin:
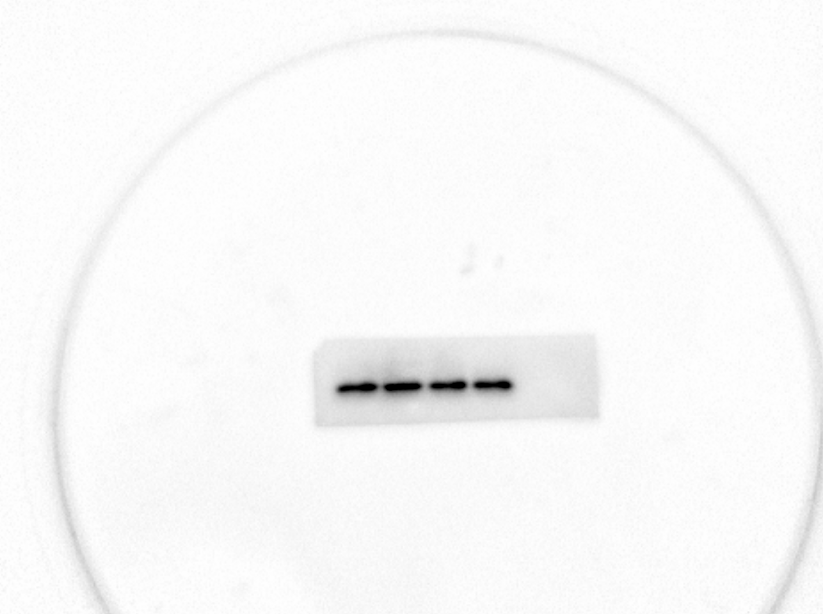


**Figure 4A**

Nrf2:
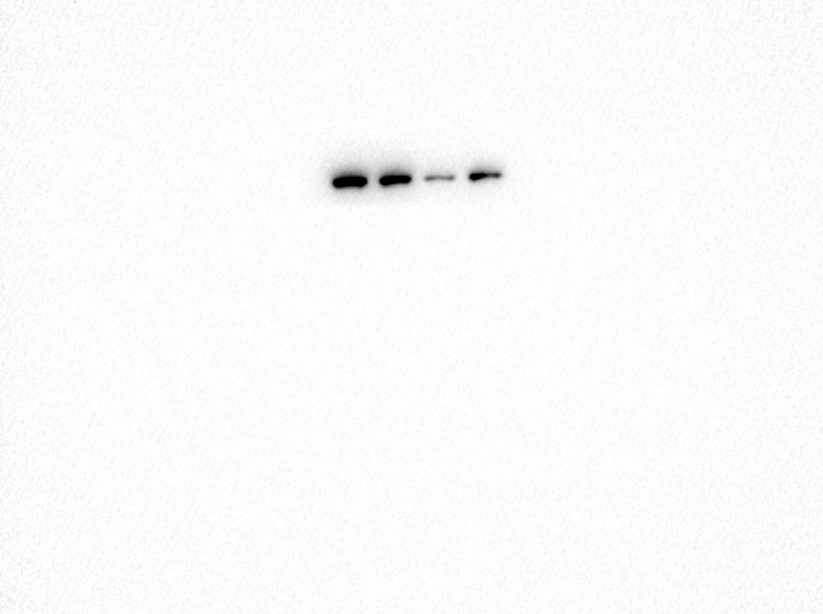


HO-1:
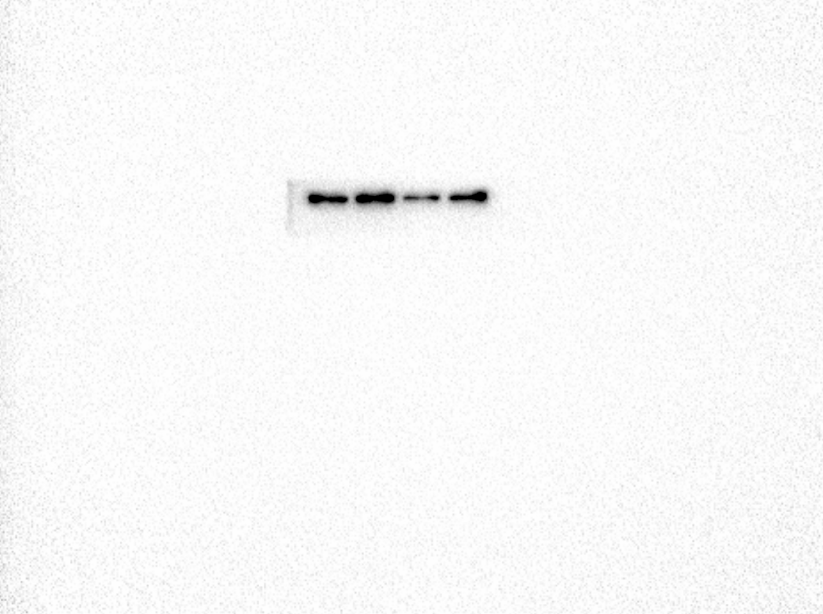


NQO1:
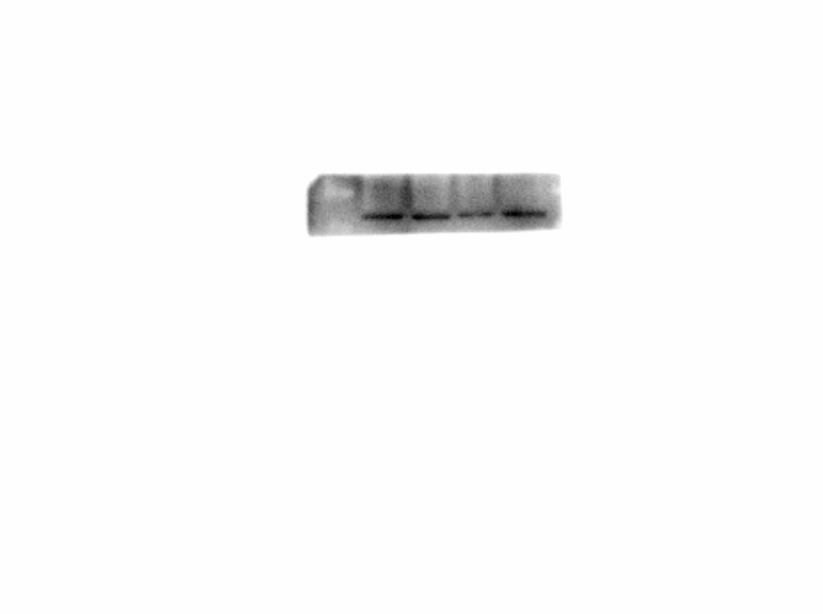


β-Actin:
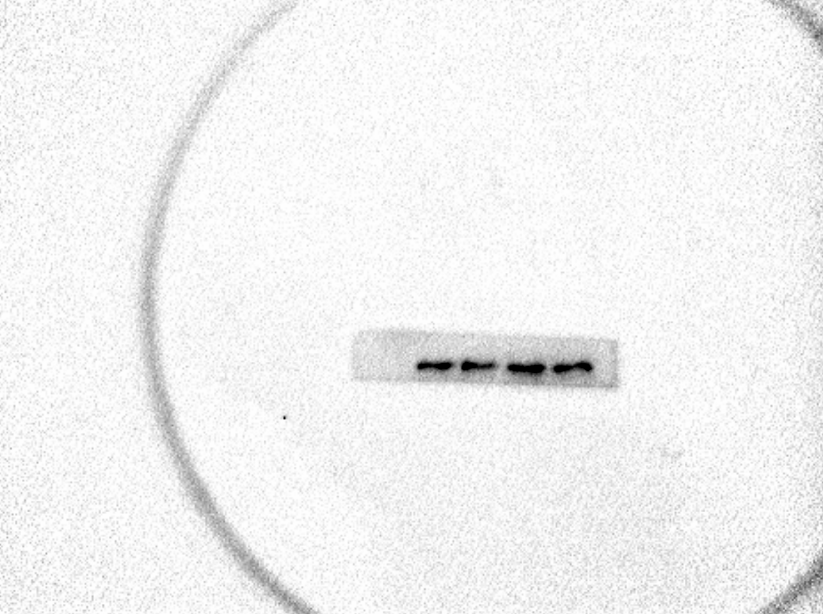


**Figure 4E**

Nrf2:
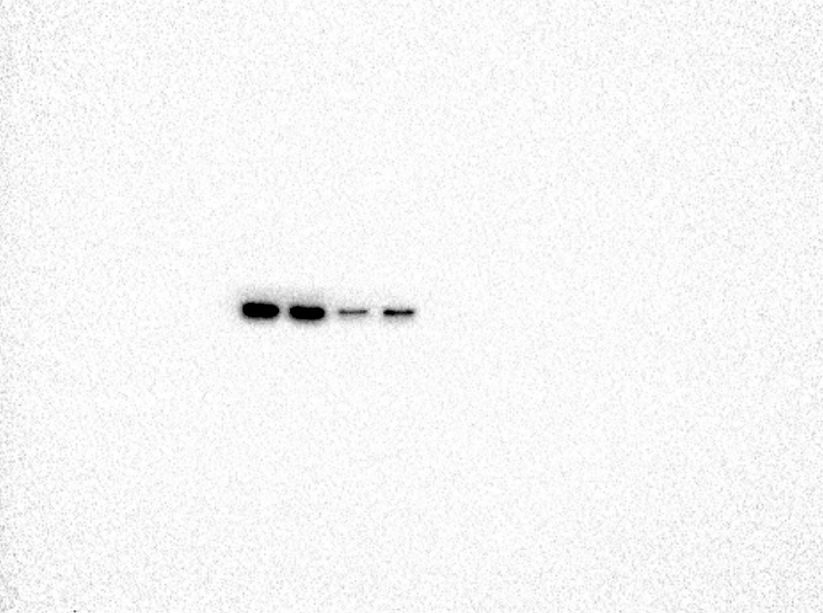


HO-1:
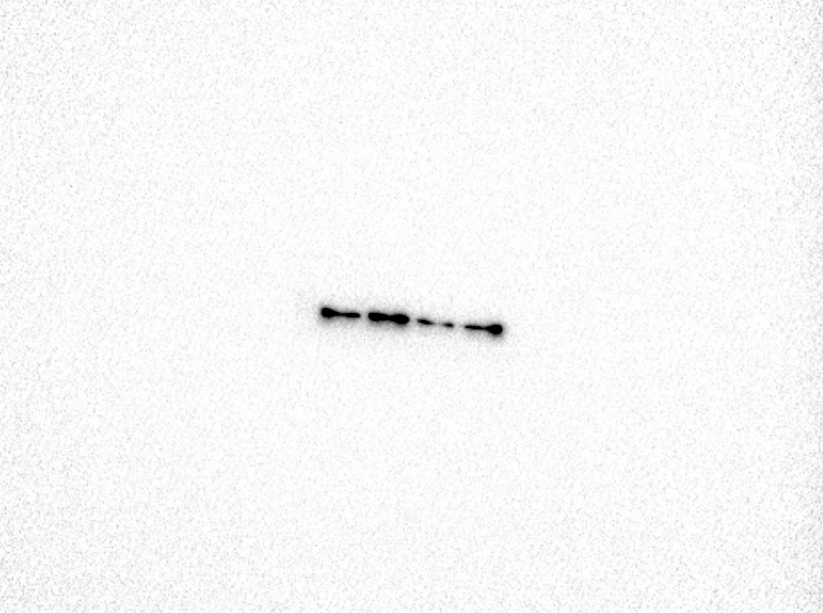


NQO1:
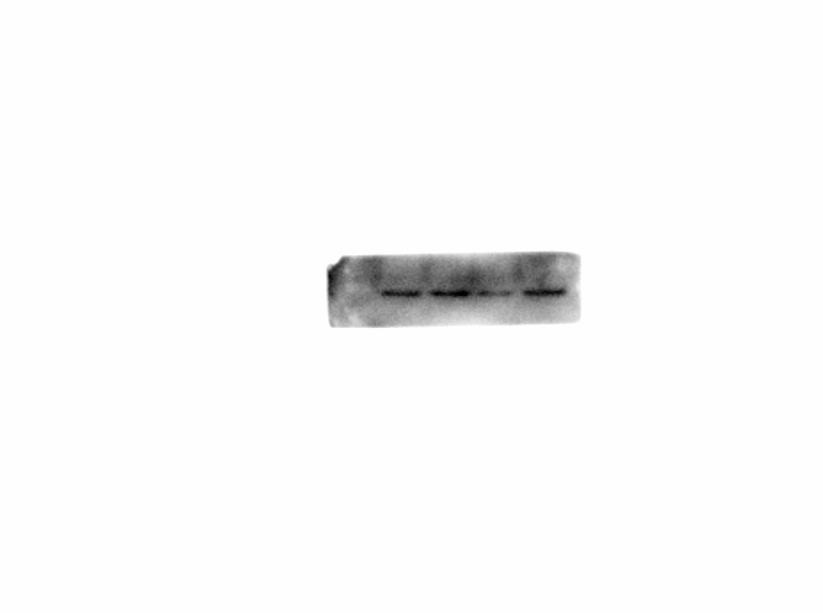


β-Actin:
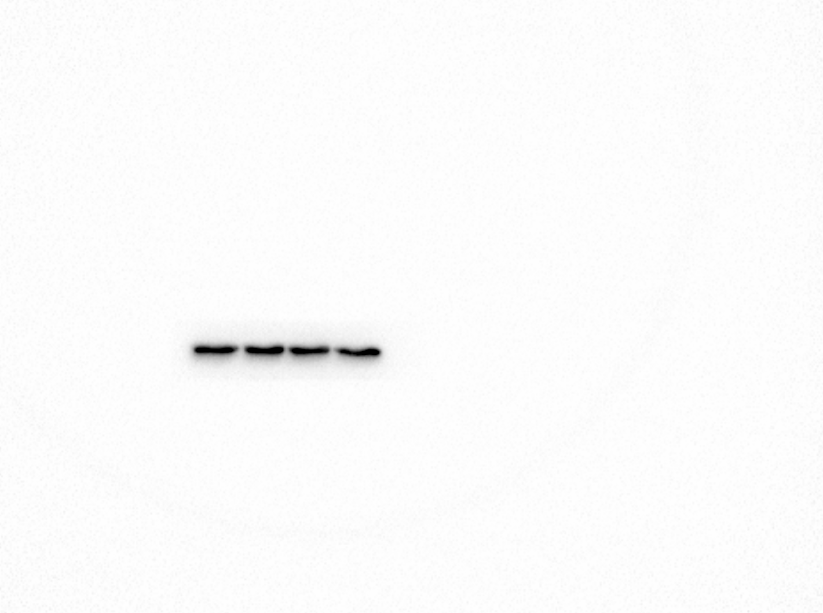


**Figure5 A**

Nrf2:
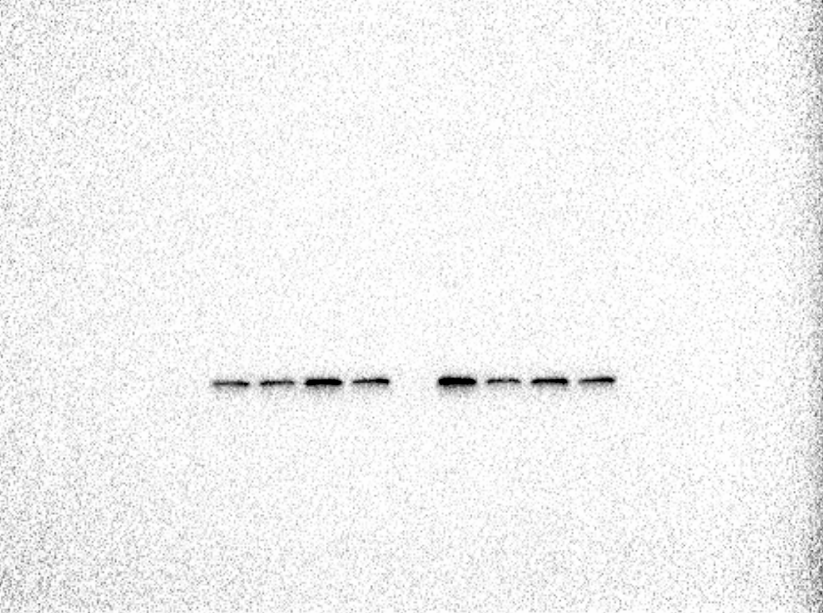


HO-1:
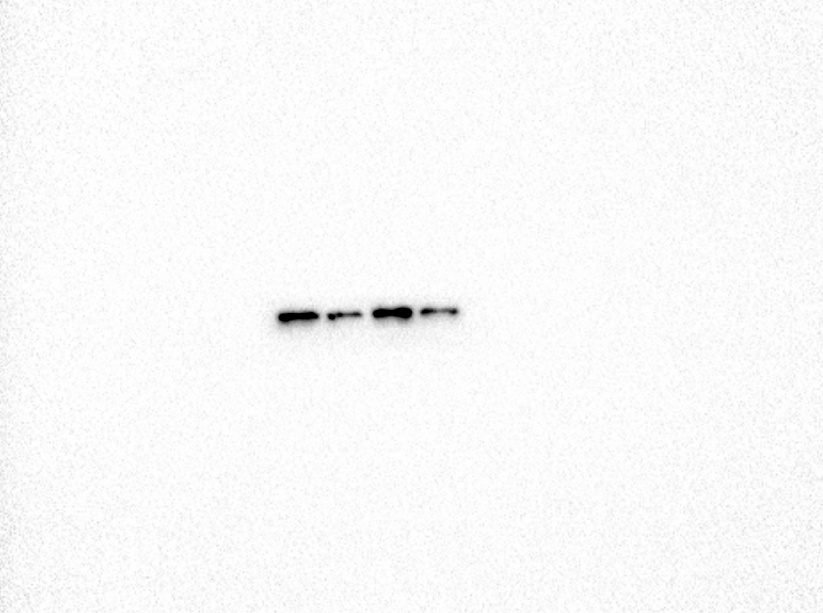


NQO1:
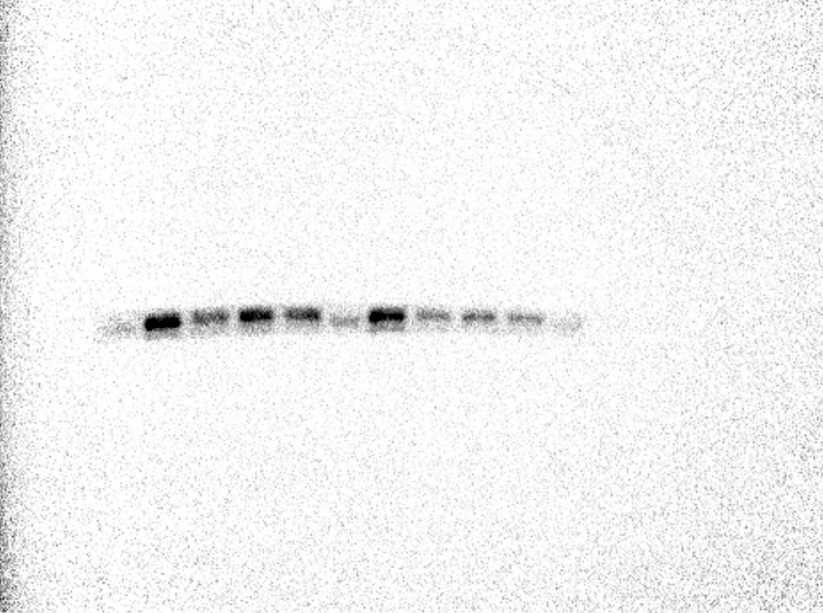


β-Actin:
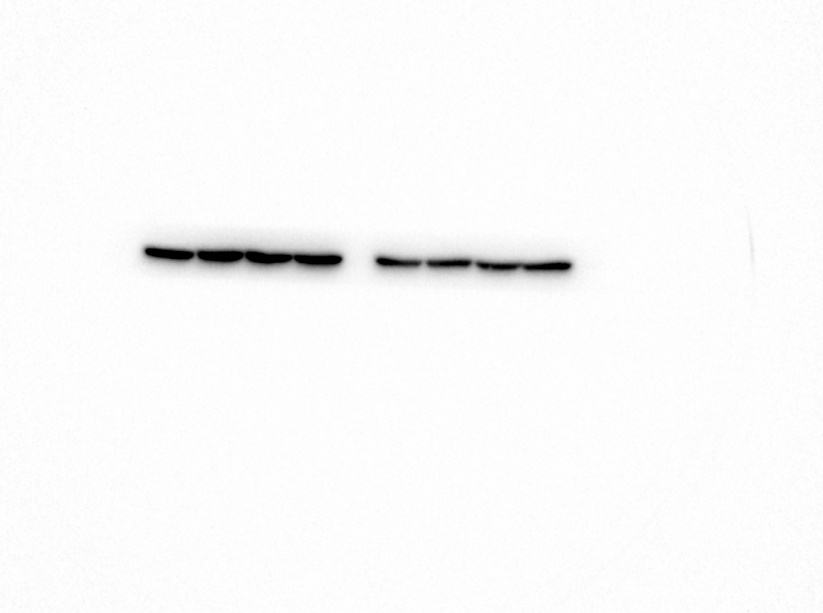


**Figure5 J**

PTGS2:
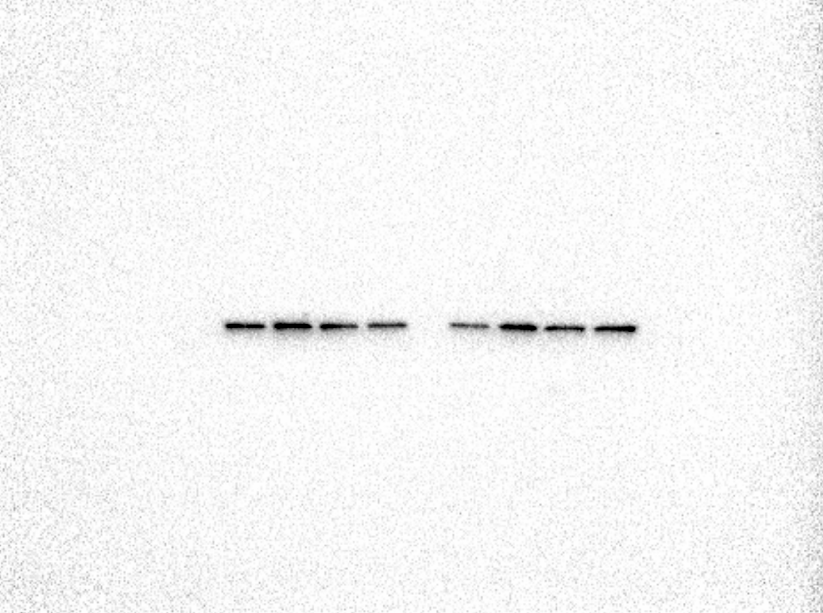


GPX4:
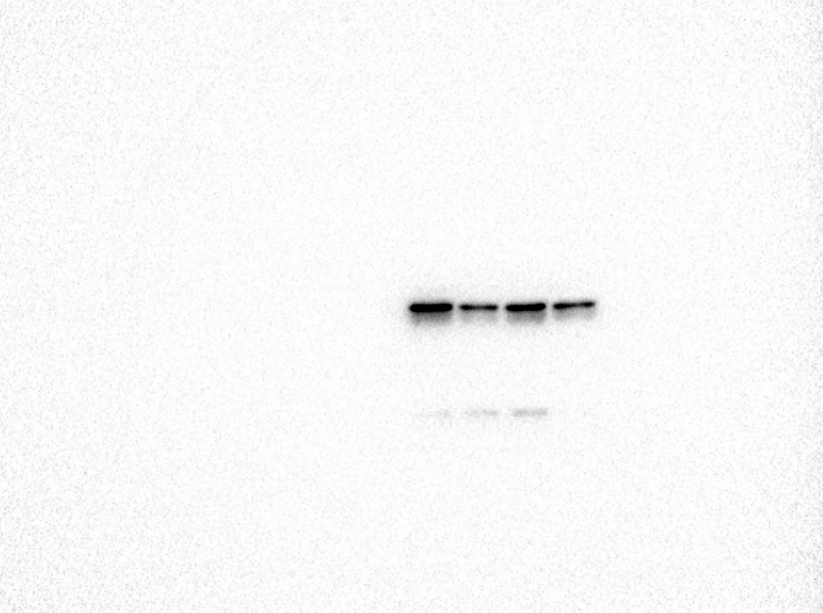


β-Actin:
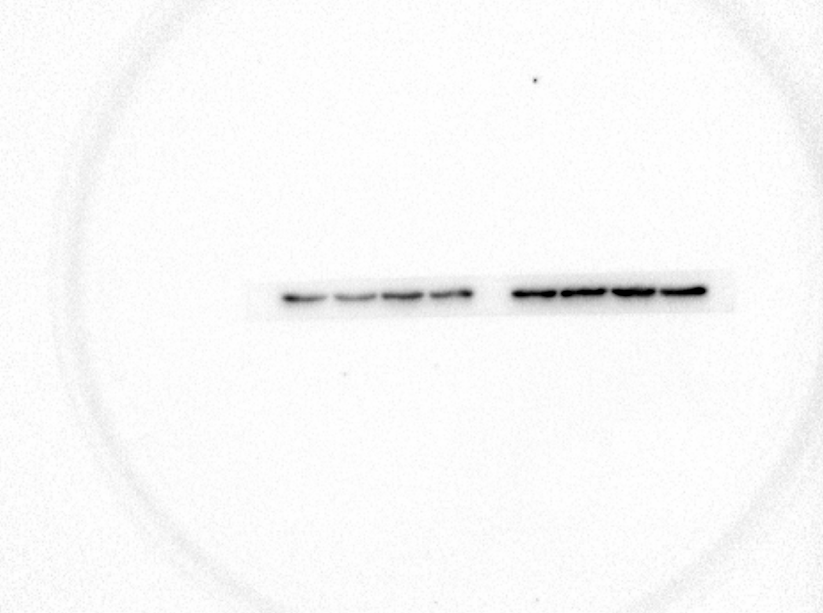


**Figure6 B**

Nrf2:
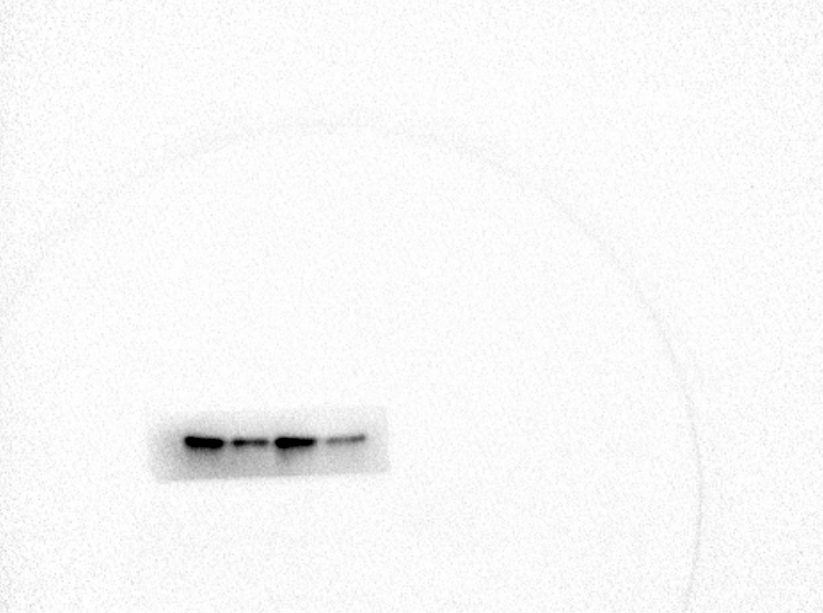


HO-1:
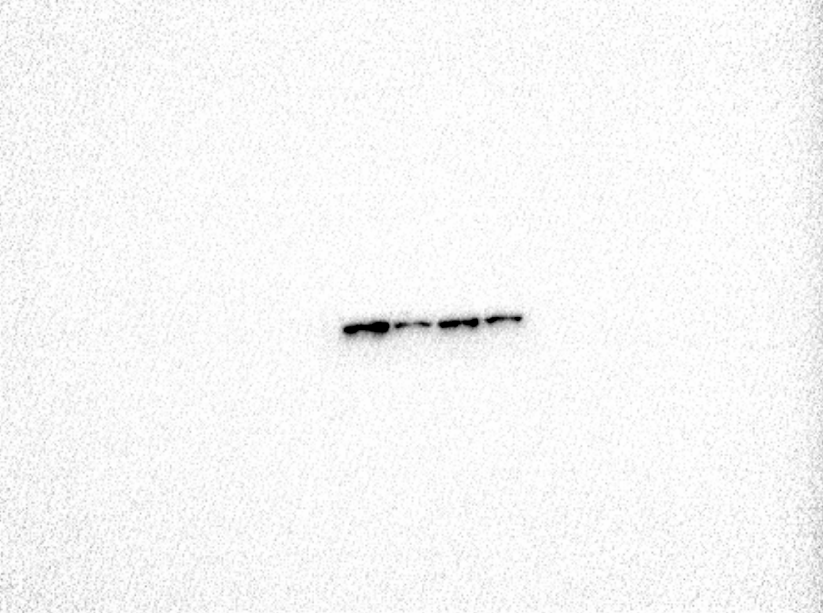


NQO1:
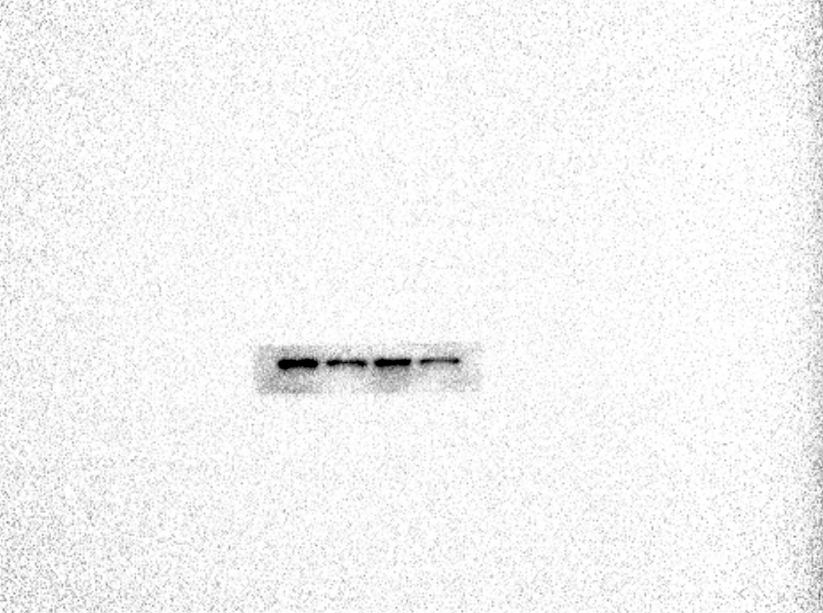


β-Actin:
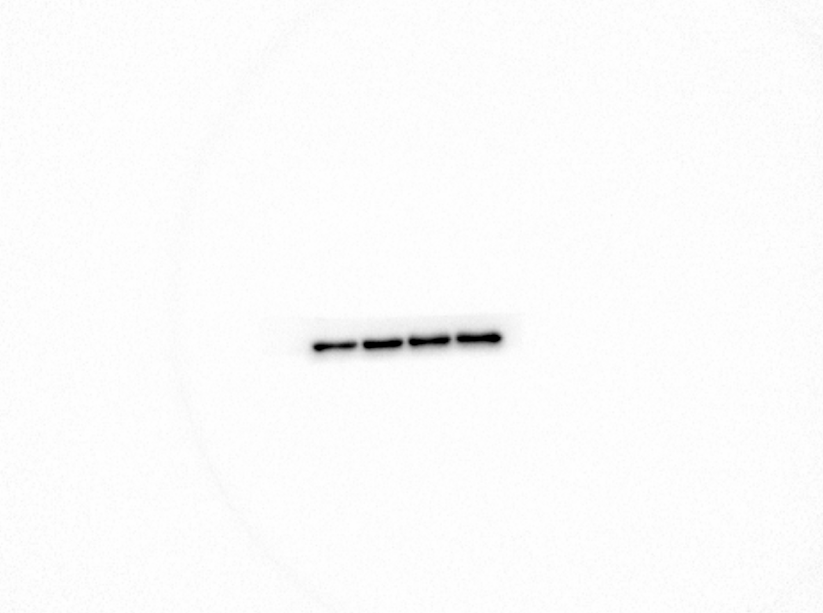


**Figure6 M**

PTGS2:
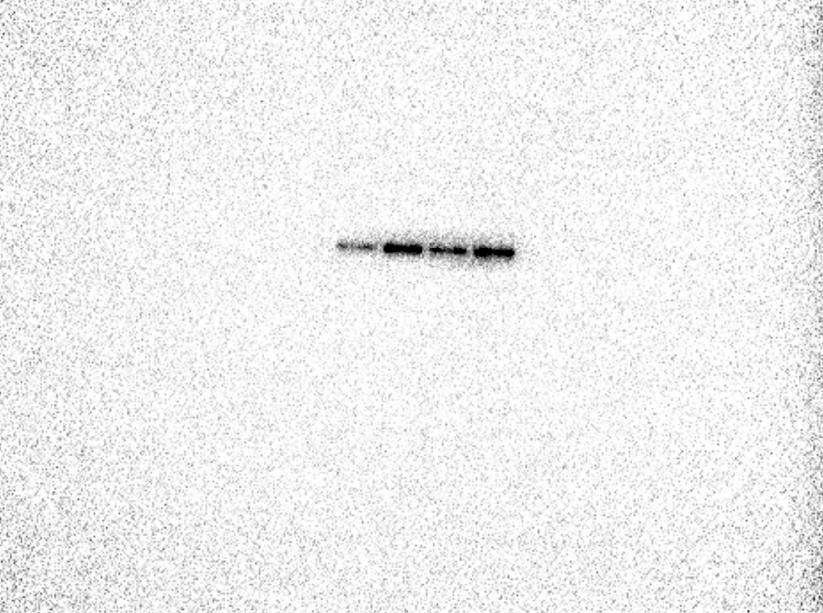


GPX4:
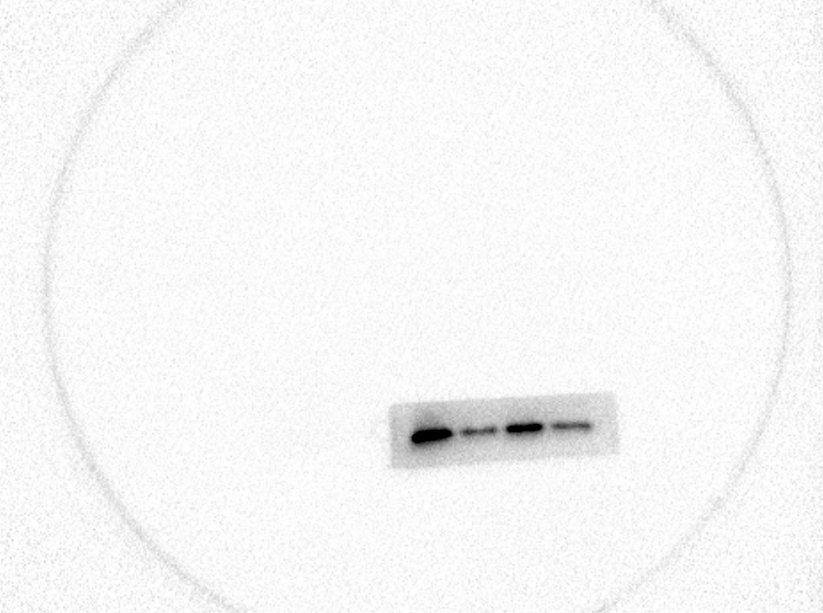


β-Actin:
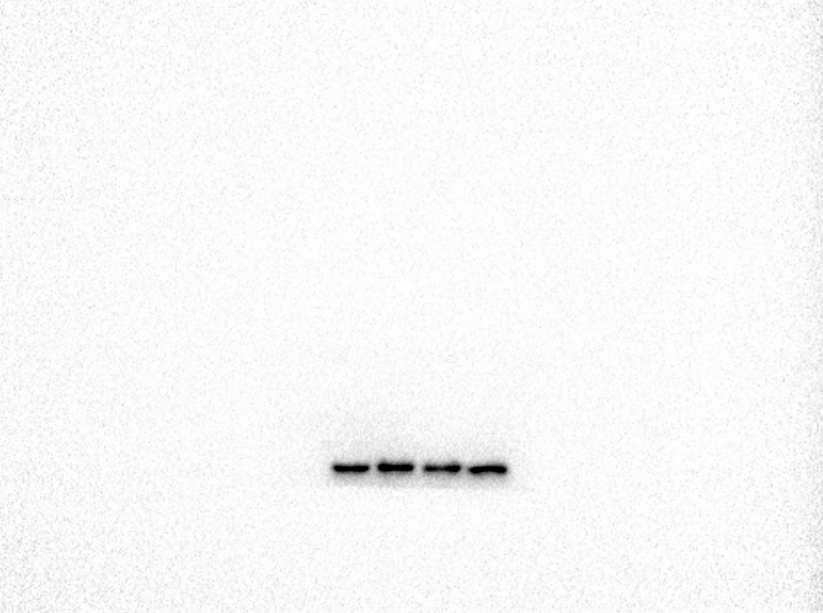

Supplement: Multimedia component 1 [file mmc1.docx]
